# Understanding facilitators and barriers to COVID-19 vaccination in the Zimbabwean population: a qualitative analysis

**DOI:** 10.1186/s12889-024-18650-3

**Published:** 2024-04-29

**Authors:** Nicholas Midzi, Masceline Jenipher Mutsaka-Makuvaza, Lincoln Sunganai Charimari, Priscilla Mangwiro, Tonderai Manengureni, Gladys Mugadza

**Affiliations:** 1 National Institute of Health Research, Ministry of Health and Child Care, Harare, Zimbabwe; 2 Department of Microbiology and Parasitology, School of Medicine and Pharmacy, University of Rwanda, Butare, Rwanda; 3 World Health Organization, Zimbabwe Country Office, Harare, Zimbabwe; 4 College of Health Sciences, University of Zimbabwe, Harare, Zimbabwe

**Keywords:** COVID-19, Vaccine uptake, Facilitators, Barriers, Qualitative

## Abstract

**Background:**

Vaccines are effective biological interventions that reduce health burdens. However, during the COVID-19 pandemic, there were concerns about varying levels of COVID-19 vaccination coverage in the Zimbabwean population. This study aimed to understand facilitators and barriers to COVID-19 vaccine uptake in the Zimbabwean population.

**Methods:**

In September–October 2023, key informant interviews (KIIs) and focus group discussions (FGDs) were conducted with a sample comprising health workers, village health workers, church leaders, traditional healers, teachers, youth leaders and the general population selected across the country. At each site, the participant sample was homogeneous. Data were collected using audiotapes, transcribed verbatim, and translated into English. Data were analysed manually using thematic analysis.

**Results:**

Ten FGDs and 30 KIIs were conducted across the country. Among the facilitators of COVID-19 vaccine uptake were the perceived benefits of COVID-19 vaccination, such as protection from infection, severe disease and death. People also complied with COVID-19 vaccination because of the government’s call for mandatory vaccination, travel restrictions, restrictions when entering some premises for services, visiting, working, learning and functions. Barriers to COVID-19 vaccine uptake included low-risk perception, negative attitudes emanating from concerns about the origins of the vaccines, COVID-19 cases or death of vaccinated people, negative peer influence, religious doctrines, cultural beliefs and misconceptions circulating through social media. Other barriers included knowledge gaps on COVID-19 vaccines, safety, effectiveness, side effects, access-related challenges to COVID-19 services and concerns over the changing policy on COVID-19 vaccination.

**Conclusions:**

The study has shown the importance of community engagement and data-driven public health communication strategies to facilitate behaviour change for increased uptake of a vaccine. In future epidemics, public health campaigns should focus on the timely provision of information explaining the benefits of an intervention, addressing safety concerns more effectively. To build trust and hence improve vaccine uptake by the public, there is a need for continuous engagement with people and to provide platforms for dialogue to address issues contributing to low vaccine uptake.

## Introduction

From 2020 to 2022, coronavirus disease-19 (COVID-19), caused by severe acute respiratory syndrome coronavirus 2 (SARS-CoV-2), became a significant threat worldwide. Following the isolation of the SARS-CoV-2 virus in late 2019, its genetic sequence was published in January 2020, triggering the development of COVID-19 vaccines [1]. Vaccines offer a window of hope as the most important public health measure and most effective strategy to protect people from the devastating outcomes of the COVID-19 pandemic [2, 3].

In Zimbabwe, COVID-19 vaccination was launched on the 18th of February 2021 in a phased approach, starting with frontline workers (healthcare workers, uniformed forces, public servants) and then the general population (≥ 18 years). The vaccination age group was later extended to include children aged ≥ 12 years. The COVID-19 vaccines that were approved for use in the country were Sinopharm and Sinovac from China, Covaxin from India and Sputnic-V from Russia. Despite the availability of vaccines, with time, the vaccination data showed a decrease in the level of COVID-19 vaccine uptake in the country [4].

Some studies have shown that there is variability in individuals’ willingness to accept COVID-19 vaccines for different reasons. In Canada, while COVID-19 vaccine acceptability was high (68%), approximately 20% of the participants mentioned that they would not receive the vaccine, and 12% were unsure of their choices. Those with high concern about COVID-19 reported that they would take the COVID-19 vaccine [5]. In Bangladesh, 26.42% of respondents were hesitant about the COVID-19 vaccine, 70.33% were either already vaccinated or were willing to get vaccinated, and 3.25% refused it definitely [6]. In the United States, willingness to be vaccinated decreased with time due to concerns about the side effects and long-term health problems, as well as doubts about the vaccine’s efficiency [7]. In some settings, doubts, mistrust, and the dissemination of misinformation have resulted in a lack of willingness to be vaccinated against COVID-19 [8]. Reliance on social media for information regarding COVID-19 has also been associated with vaccine hesitancy, likely due to the widespread misinformation circulating on the platform [9–11].

On the 5th of May 2023, the World Health Organization lifted the public health emergency of international concern (PHEIC) status for COVID-19 [12]. However, the risk remains of new variants emerging, causing new surges in cases and deaths; thus, it is imperative to transition to long-term management of the pandemic, such as scaling up vaccinations such as primary and booster COVID-19 vaccination to all vulnerable populations [12, 13]. To support these activities, there is a need to understand the barriers and facilitators of adherence to public health interventions [14] to inform public health response and communication strategies. With scarce data about factors that affected compliance with COVID-19 vaccine uptake in Zimbabwe, this study was undertaken to understand drivers and barriers to COVID-19 vaccine uptake in the country. The information is of critical use for drawing lessons on how to deal with future surges or pandemics.

## Methodology

This is a sub study of a larger study that sought to understand social-behavioural determinants of population compliance with public health and social measures (PHSM) and COVID-19 vaccine uptake in 6 selected African countries coordinated by the World Health Organization - Regional Office for Africa.

### Study setting and population

Eight out of 10 provinces were selected for the qualitative study. Due to limited budget, it was not feasible and logistically manageable to collect data in all the provinces. In each of the selected province, a district with low vaccine uptake as recorded in the District Health Information Software 2 (DHIS2 database) was selected for the study. To capture diversity in perceptions, behaviours, experiences and recommendations from the community on COVID-19, different social and economic population groups comprising of health workers, village health workers, teachers, traditional healers, transporters, religious leaders, women leaders, youth leaders, and the general population were selected across the country (Table 1). A homogeneous population group was chosen for both the focus group discussion (FGD) and key informant interviews (KII) at each site to allow for data saturation with a sample of participants that is manageable, enhancing smooth coding and generation of themes.

**Table 1:** Study sites and their COVID-19 vaccine coverage as of end of june 2022

Province	District	Study group	% COVID-19 vaccination coverage		
			1st dose	2nd dose	3rd dose
Harare	Epworth	Women Leaders	47.1^*^	35.2^*^	5.5^*^
Harare	Mbare	Transporters			
Chitungwiza	Zengeza	Religious Leaders	54.6	31.0	4.4
Mashonaland Central	Rushinga	Youth Leaders	43.1	27.1	1.0
Masvingo	Chiredzi	Traditional Healers	32.0	30.9	4.5
Mashonaland West	Makonde	Village Health Workers	40.0	24.9	5.0
Matabeleland North	Binga	Health workers	35.7	35.7	4.3
Matabeleland South	Insiza	General Population	50.9	37.2	6.1
Midlands	Gokwe South	Teachers	42.5	22.3	3.7
Mashonaland East	Seke	General Population	36.7	27.8	3.7

* The vaccination coverage includes Epworth and Mbare districts. The vaccination coverages for all districts in Harare are combined in the District Health Information Software 2 database. Thus, the vaccination coverage for Epworth and Mbare districts could not be separated. Table adapted from Midzi et al. [15]

In Makonde district the study was conducted at the growth point, which was previously a farming compound. Most of the households had a functional tippy tap popularly referred to as *chigubhu gear* among the locals. The study was also conducted at a growth point in Binga. The area which is a resort business area attracting many tourists and fish traders, has a mixed culture community but mainly dominated by the Tonga tribe. Most of the business facilities had hand washing facilities or sanitizers by the entrance. In Insiza district the study sites were the growth point and rural area. Study participants and people visiting the clinic were wearing their masks properly. At the rural clinic where the FGD was conducted, there was no sufficient water supply, no convenient hand washing facility at points of entry and the Blair toilets were not well maintained. One of the study districts, Seke is a peri-urban area of Harare. Most of the people commute to Harare for work and school. Zengeza district is a high density urban setting of mixed cultures located in Chitungwiza, a dormitory town for Harare metropolitan city. Residents have access to different media sources. There is shortage of water supply and this result in many people converging at boreholes for water collection. Mbare district is an over populated high-density suburb close to Harare central business district. The area which houses mixed cultures is characterized by vending, trading and it also houses a central bus park station for transport connecting to different cities, districts and across the borders of Zimbabwe. Epworth district is a peri-urban densely populated area with a mixture of cultures and ethnic background. The area has a lot of illegal housing structures with compromised water supply, scarce hand washing facilities and ablution facilities. There is a lot of business going on facilitating interaction between Epworth and the Harare central business district. In Gokwe South, the study site was at a growth point. It was observed that the population including the school children was not wearing face masks, not observing physical distancing and there were no visible hand washing facilities at entry points. The study site in Rushinga district was a growth point. The district borders with Mozambique and has mixed cultures. Most of the shops were observed to have hand washing facilities. In Chiredzi district, the study was conducted in town. There is a mixed culture population in this town and it also supports a lot of trading between Harare, Chiredzi, Masvingo and South Africa. The town is densely populated with a lot of interrupted water supply.

### Study design

A descriptive phenomenology qualitative study was conducted in line with the standard consolidated criteria for reporting qualitative studies [16].

### Sampling procedure and sample size

Purposive sampling was used in which participants who shared the same characteristics and had the potential to provide rich, relevant and diverse data pertinent to the research questions were selected per site (Table 1). Overall, participants were considered based on the feasibility of accessing and recruiting them, their availability and willingness to sign the informed consent and participate in the study. Key informants were chosen based on: (i) having first-hand information of their community, fellow residents, and issues about COVID-19 based on their experience, professional expertise, special social positions, current or previous participation in a COVID-19 program in the area (ii) being non-judgmental and sensitive to differences among community members. At each site, 3 key informants were identified for the key informant interviews. In total, 30 key informant interviews were conducted. FGD participants were selected on the basis that they were able to express themselves in a group setting to provide diverse perspectives and experiences related to COVID-19 in their community. One FGD was conducted per site (Table 1). In total, 10 focus group discussions were conducted across the country. Each FGD comprised 10 participants; thus, 100 participants were included in the FGDs in this study. Generally, 4–12 participants are recommended per FGD [16].

### Study guides and data collection

The study guides were developed by the World Health Organization, with topic guides being informed by literature on the uptake of vaccines. The study guides were conceptualized in the Zimbabwean setting by the local researchers. The guides consisted of a semi-structured interview guide for KII, focus group discussion guide and observation checklist. These were uniformly used to collect data across all study groups. The interview and FGD guide had similar questions seeking to understand the participants and community general knowledge and experiences on COVID-19, recommended COVID-19 preventive measures, challenges faced by the community hindering compliance to COVID-19 preventive measures, influencers promoting uptake of vaccines and other preventive measures in the community and recommendations to improve compliance to vaccination and other preventive measures in the community. The observation checklist was used to record key features such as location of study site (i.e. market place, church, public place, and homestead) presence of hand washing facilities/sanitizers, whether people were washing their hands, correct wearing of face mask and whether people were practicing physical distance at the study sites.

The research team comprised a PhD expert in grounding theory studies and ethnography, PhD holders in community health, individuals with master’s in public health and research assistants with at least a degree in social sciences or Public Health. All the FGDs and the KIIs were conducted physically. Sensitization and mobilization of the community were conducted by the Environmental Health Officers. The cadre visited the meeting points for familiarization, sensitization and mobilization of the community before the day of the study. Each FGD took an average of 45 min, while the KIIs took approximately 30 min each. The KIIs and FGDs were conducted either in English or any of the two local languages, Shona or Ndebele, depending on the language spoken in the province and district selected for the study. Each session was led by a skilled moderator while the assistant moderator observed and took notes. With consent from participants, interviews and group discussions were recorded.

### Data analysis

All the audio recordings were transcribed verbatim. Sessions that were conducted in the local language were transcribed verbatim and translated into English. The translation were conducted by the moderator and co-moderator after data collection at each site. These personnel had experience in bilingual translation and proficient in both the original language of the script and English. The translated scripts were reviewed by a second language expert to ensure accuracy and consistency of the original translation. Using the thematic analysis approach, an analytic template, aligned with the FGD and KII guides, was developed as a starting point for analysis. Coding of the transcripts was initially conducted by an experienced qualitative researcher. The other investigators made input to the codes based on the data set, cross-checking with the transcripts. Subthemes were identified by initially clustering and sorting out the codes, categorizing them according to the meaning and trend of ideas. The main themes were derived from the subthemes based on the relevance to the research question and extracts of the transcripts. The most important and repetitive quotes were chosen to represent the selected themes. The research team took time to discuss the analysis process and review, name and define themes. No software was used in the analysis.

### Credibility and trustworthiness

To enhance credibility of the study, the researchers considered diversity by sampling different population groups of different demographic characteristics (age, social status, cultural norms, religion, economic status and area of origin), with purposive sampling to remove selection bias inherent in already existing population groups. The researchers applied the circling reality principle by obtaining information on the same study variables from different study groups. Triangulation was also done through data sources (KII and FGDs) with a similar questions on the interview and FGD guides to enhance dependability. The researchers tried to enhance honesty and opening up among participants by establishing a good rapport, trustworthiness and assuring them of confidentiality. To increase transferability, participants were quoted directly and a complete description of the entire process is given.

## Results

### Study participant demographics

Out of the estimated sample size of 130 participants comprising 30 participants for KIIs and 100 participants for FGDs, 128 (98.4%) took part in the study. Two participants were lost to follow-up in 2 FGDs: one for traditional healers and the other for women leaders. Among the participants, 70 (54.7%) were males, and 79 (61.8%) had attained a secondary level of education. The average age of the study participants was 46 years (SD = 11), and the age range was 20–72 years.

### Facilitators and barriers to COVID-19 vaccination

Two themes identified as facilitators of COVID-19 vaccination were perceived benefits of the vaccines and triggers for compliance with vaccination. Six themes identified as barriers to uptake of the vaccines included; knowledge gaps on COVID-19 vaccines, no risk or low risk perception, negative attitude towards COVID-19 vaccination, access-related challenges, concerns about COVID-19 vaccine side effects, safety and effectiveness, and changing policies on COVID-19 vaccines (Table 2).

**Table 2:** Thematic findings from the study

Category	Theme	Codes
Facilitators	Perceived benefits of the vaccines	Reduces the number of COVID-19 cases, vaccines are protective, death toll reduced, they boost immune system, COVID-19 illness will not be severe, prevents spread of disease, greater chances of survival, and assured safety after vaccination.
	Triggers for compliance with vaccination	Availability of information, encouragement from community leaders, availability of mobile outreach, stipulated mandatory vaccination by tertiary institutions, companies and government, desire to attend functions like church services, and birthday parties where there were asked to provide vaccination cards, to reduce the pandemic burden, ease of traveling within or outside Zimbabwe and feeling of being relieved and death of a loved one.
Barriers	Knowledge gaps on COVID-19 vaccines	Limited information on what vaccines do to our bodies, lack of education, lack of knowledge by church leaders, elderly would take time to understand about the vaccine so this delayed the process, people were depending on rumours and social media, social media sent false information, different types of vaccines available made people skeptical.
	No risk or low risk perception	COVID-19 is not there, selling of vaccination cards, elderly are at risk.
	Negative attitude towards COVID-19 vaccination	Fear of the unknown on vaccines, lack of interest, lack of conviction, negative information on vaccination, social media seemed a bit exaggerated, resistance to the information, negative peer influence, outdated and strange beliefs especially the elders, fear of getting vaccinated, religious sect doctrines, vaccination linked to Satanism, people perceive COVID-19 as money making scheme and not a health-related issue, information that people with hypertension and diabetes not to be vaccinated, fear of death, trust issues, vaccines are meant to depopulate the communities.
	Access-related challenges	Transport challenges to carry nurses to vaccination points closer to communities, long distance to the nearest vaccination centre/ health centre, no transport money, some people are too old to reach the clinic or even the vaccination point, poor or loss of radio transmission result in missing vaccination, Second vaccine dose was scarce, One had to receive a 2nd dose at the same center they were vaccinated even after relocation.
	Concerns about COVID-19 vaccine side effects, safety and effectiveness	Vaccines were hurriedly produced without much evidence, vaccines causes blood clots, people with chronic conditions experienced many side effects, infertility, adverse reactions to the vaccine such as itching, vaccines kills, after 2 years, vaccines monsters, vaccines interfere with family planning methods, reduces men’s ability to have sex.
	Changing policies on COVID-19 vaccines	Inclusion of children into vaccination programme, vaccination of pregnant mothers,

Some individuals started appreciating COVID-19 vaccines when they noticed a decline in the number of COVID-19 cases after the introduction of the COVID-19 vaccination programme. Most participants were aware that COVID-19 vaccines would boost their immune systems and protect them from COVID-19 infection. Those who became infected after vaccination presented with mild/moderate disease compared to severe infections, which were more common before the introduction of vaccines. They believed that COVID-19 vaccines reduced COVID-19 deaths, with most people recovering from COVID-19 illness due to the protective effect of the vaccines, while there was a high death rate during the pre-COVID-19 vaccine era. The following quotes illustrate how participants viewed the benefits of the vaccines.

*“Vaccines are important that they boost you, those who got infected after vaccination did not get severe disease like those who got ill in the first days of the disease. This [vaccination] reduced death so much and that helped us a lot. I hope we will get to a stage where we all get vaccinated and protected. We all got ill let me say that, no one was spared in this country, we all got ill, were bedridden and we recovered, as a result of these vaccines” (FGD participant 2, Gokwe South District, school teacher).*

*“From the moment people accepted being vaccinated for various reasons, the cases dropped, and those that were attacked most of them recovered compared to when people were attacked before being vaccinated ----” (KI 2, Gokwe South District, schools inspector)*.

Those who understood COVID-19 through education were forthcoming to be vaccinated. Those who did not believe in COVID-19 vaccinations changed their mind-sets after receiving education on COVID-19 vaccines.

*“People in our communities now understand COVID-19 better than in the first days of the disease as they are now getting COVID-19 vaccination and they are now getting all three doses. Some people come on their own to the clinic to ask about COVID-19 and get vaccinated” (FGD participant 3, Insiza District, community member).*

In some communities, the health personnel and village heads were pivotal in the dissemination of COVID-19 information, encouraging people to follow preventive measures and would mobilize the community for vaccination in line with the schedule for the mobile vaccination teams. The convenience of accessing the COVID-19 vaccination teams near their households also triggered some people to be vaccinated.

“*Village health workers also have time to call meetings through village heads to inform community members about COVID-19 and the dates for mobile vaccination and mobilize people so that when nurses come, they find people ready to be vaccinated. Mobile vaccination helps reduce distance; some people simply don’t want to walk long distances, so they get vaccinated when mobile vaccination people come” (FGD participant 3, Insiza District, community member)*.

In the absence of health personnel, community leaders and nongovernmental organizations used their political, religious, social or traditional gatherings to spread messages regarding COVID-19 vaccination. They also led by example by getting vaccinated themselves. This improved the community’s attitude and motivation to be vaccinated.

*“In our locality, our MP [Member of Parliament] for Makonde constituency played a pivotal role in cascading information regarding COVID-19 prevention and this he did whenever there is a political gathering, The MP encouraged the local people to get vaccinated and led by example as he was vaccinated” (FGD participant 7, Makonde District, village health worker)*.

Vaccination was made mandatory at learning institutions or workplaces. Proof of vaccination was also used as a permit to enter some buildings or offices for official business.

*“Like here, offices there in the provincial level you cannot get there without a vaccination card, so we also encourage people to be vaccinated. Sometimes those are things that we can do to make people vaccinated. I will give you an example of nursing, nurses there was a time when we suffered from COVID-19; there is an allowance that you are given, so you can’t get an allowance without a vaccination card” (KI 1, Binga District, health worker)*.

*“---- I only got vaccinated due to work policies about COVID-19 vaccination. I also feel that most people get vaccinated because of external pressures” (FGD participant 8, Zengeza District, religious leader).*

Despite individual protection, people were triggered to get the vaccination because they believed that vaccines were important in proffering immunity to the whole country through herd immunity, thus leading to the lifting up of other preventive measures. Those who travelled locally or internationally for different reasons, such as transporters, cross-border trading, and evangelism, considered vaccination as a gate pass since most countries were now demanding proof of vaccination before someone entered their territory.

*“These vaccines are very important in that they enable the protection of people against the virus as one large body herd immunity. Second, being a travelling person, I realized that visiting other countries without being vaccinated against COVID-19 is not possible. I realized that being vaccinated against COVID-19 made my travelling experiences easier and trouble-free. When we were at airports and border posts, we did not face any constraints upon showing authorities our vaccination documents” (KI 2, Zengeza District, pastor).*

However, there were also COVID-19 vaccine refusals attributed to a lack of information on COVID-19 vaccines. Some communities felt that they did not have enough education on these vaccines before their roll-out. People were accustomed to the long process that is taken in the manufacture of vaccines in general. The accelerated manufacture of COVID-19 vaccines has resulted in many concerns about their safety and side effects in the general public. This was compounded by the discussions that were being made by medical researchers and disinformation from social media. Additionally, the different origins of the vaccines instilled confusion in people.

*“There wasn’t enough education for the people. Normally, vaccines take time to be prepared. --this was different with COVID-19 vaccines, at least ten years [is needed]. People did not know the effects, so some would shun the vaccines. Medical researchers also had arguments against the vaccines. Most people could not embrace the vaccines due to some of the information that was circulating on social media. There are also controversies around the different vaccines used across the world” (KI 1, Seke District, development economist).*

Some people were confused about the benefits of vaccination since they were told to continue wearing face masks after being vaccinated. Due to a lack of education, some people had questions about why they should be vaccinated at an older age when most vaccines are given when an individual is still young. Noting that many deaths were recorded in western and eastern countries, some people thought that getting vaccinated would make them vulnerable to death due to COVID-19. Some people could also not get vaccinated because they had a belief that Africans had a strong immune system compared to white people; thus, they would be able to fight off the disease without vaccination.

*“------ there is only one challenge with these vaccines, especially in Africa that is getting vaccinated at an older age. The other question people asked was, does the vaccine work? This was because after getting the injection, people would be told to continue wearing face masks. Therefore, people question the benefits of getting vaccinated. --- if you look at countries like the United States, many white people died, so people here in Africa were saying that this pandemic was only going to affect white people in the United States or the Chinese people. So getting vaccinated here in Africa would make us vulnerable to death like what the Americans and Chinese are doing. We Africans are immunologically strong compared to whites—” (KI 1, Zengeza District, pastor)*.

Some individuals had the misconception that the COVID-19 vaccines would lead to death after a certain period, while some thought it had negative effects on those with comorbidities. Rumours were also circulating that this was another family planning method, and it reduced the man’s ability to have sex among other effects. The community did not get clarity from the responsible authorities regarding these rumours. The people’s fears are illustrated as follows:

*“Most people were misinformed about the vaccine. Some thought that it would trigger a chronic illness ending in death after ten years, for example. Some also thought that if you had comorbidities, you would be compromised, leading to adverse events and possible death. A lot of fear surrounded the aspect of being vaccinated” (KI 1, Chiredzi District, traditional healer)*.

*“---- A lot of people were complaining saying it is another way of family planning methods, they were complaining that it reduces the men’s ability to have sex, one lady approached us saying she had been bleeding for 2 months since she got vaccinated. So they would also be telling each other that nothing would happen in the bedroom. There were no answers for that; hence, we were not equipped to answer” (FGD respondent 3, Binga District, health worker)*.

Multiple disease interventions in recent years have also resulted in community fatigue. Some communities now had the belief that they were being used as guinea pigs.

*“The number of programs coming up has been a lot, we had MDA, for bilharzia, for trachoma, so every other month we have a mass drug administration or vaccination coming. Communities were tired and for this community, they would think that the program was coming to them as experiments since they are a minority group” (FGD respondent 8, Binga District, health worker)*.

Population groups who perceived to be at low risk had an inner conviction that COVID-19 did not exist or that they were immune to COVID-19 illness; hence, they declined to take up COVID-19 vaccines. Some elderly people thought that COVID-19 did not exist and that the current disease was just influenza but had been given a new name. Thus, they did not consider themselves to be at risk and hence refused to be vaccinated.

*“Some local people say there is nothing called COVID-19, and they declined COVID-19 vaccination. Some elderly people say COVID-19 is a new name for a condition that has been there for ages which was called influenza” (FGD participant 10, Seke District, community member)*.

Some people had no tangible reason to refuse COVID-19 vaccination. Some people had a negative attitude towards COVID-19 vaccines because they had witnessed COVID-19 cases in vaccinated individuals. They believed that similar to other previous viral diseases, COVID-19 would end on its own. There was also a myth that the COVID-19 vaccines were meant to reduce the population in the communities.

*“Some people don’t get vaccinated because when you are vaccinated you still get infected by COVID-19 so it’s pointless to get vaccinated since you get the disease whether vaccinated or not. People say COVID is flu and it will end with time just like Frazer. Some people don’t get vaccinated because there is a rumour that the vaccine is meant to depopulate the communities” (KI 1, Insiza District, traditional chief)*.

Social media contributed largely to the shunning of vaccination by the public, as the information it provided seemed slightly exaggerated. After the introduction of the vaccine, despite positive uptake in the initial phases, a decline in uptake was witnessed as negative feedback started filtering through social media from those who were vaccinated. This disinformation instilled fear in people, resulting in negative attitudes towards the vaccines.

*“Another attributing factor towards a decline in the uptake of COVID-19 vaccination was due to the negative effects of social media where people gave such testimonies as when I got vaccinated, I had a stroke, some said my breast was enlarged, some said our relatives died after vaccination. Such testimonies instilled fear in the local people, and they shunned the vaccination exercise” (FGD participant 2, Makonde District, village health worker)*.

Some religious doctrines that prohibit vaccination influenced the congregants’ attitude towards COVID-19 vaccination in the Zimbabwean population. They placed more emphasis on the use of lemon and milk as natural remedies for managing the COVID-19 pandemic.

*“In some areas where there are religious sects of the Johanne Marange who still hold the belief that they don’t seek medical treatment and get vaccinated, ----. I have a relative who is a member of the Johanne Marange Apostolic sect who ended up resigning because he refused to be vaccinated even when the company policy needed all employees to be vaccinated. He would say God will protect me and not the vaccines” (KI 3, Makonde District, village health worker)*.

People started selling COVID-19 vaccination cards, and since these were requested at entry points, people thought that having one without being vaccinated was enough

“Selling of COVID-19 cards stood as a barrier to getting vaccinated against COVID-19” *(FGD participant 1, Seke District, community member).*

In as much as some people declined vaccination, some were willing but faced challenges in accessing such services. When the vaccination programme started, the COVID-19 vaccines were in short supply. Those who visited the health centres for vaccination would sometimes be turned back without receiving the vaccine, be it the first dose or second dose due to the unavailability of the vaccine. The expectation by the Ministry of Health that an individual should complete vaccination in one place also posed challenges for travellers. All these challenges resulted in some people not being able to complete their vaccination doses. Although efforts were made to provide COVID-19 mobile vaccination teams in the community, in some communities, it was stated that not all people could access these mobile teams. Some had to walk long distances to the health facilities to get the vaccine, while others, despite the distance, had walking challenges and had no transport or transport money to get them to the nearest vaccination facility.

*“----when we started using vaccines, the major challenge was the availability of the vaccines. People would be returned home when they visited the clinic for vaccination. At times, one will be injected with the first dose but will not find the second dose. Another challenge was the law around vaccination. If a person is vaccinated in Mutare, they have to finish all the doses in Mutare. This was a problem since others would relocate. Some will end up not completing the doses. They would argue that l am better off than those who didn’t even receive the first dose. Accessibility was another challenge. There was a mobile vaccination team, but they could not access everyone. Therefore, people in those areas had challenges accessing vaccines” (KI 1, Seke District, development economist).*

At some vaccination centres, there were very long queues, and people would wait for a long time before being attended to, which discouraged other people from being vaccinated.

*“--------. By and large, most vaccination centres had very long queues that tended to discourage those willing to receive the vaccine---” (FGD participant 8, Zengeza District, religious leader).*

Some individuals stated that they experienced some side effects after vaccination. There were also social media reports of persistent and non-persistent side effects, such as infertility, itching, sexual dysfunction in men, amenorrhea in women, blood clotting, hypertension, heart conditions and death, possibly within 2 to 3 years post-vaccination.

*“People were vaccinated, but many people complained of side effects that ranged from sexual dysfunction in men to amenorrhea in women” (FGD participant 7, Seke District, community member).*

Some deaths in the community of those who had comorbidities raised concerns about the safety of the vaccines in that population group.

*“--- Some hypertensive people were vaccinated and died possibly due to reasons other than COVID-19 itself. Amidst the explanation given to the people without explanation on the possible causes of deaths, these incidents made people condemn the vaccine and instilled fear in the local people” (FGD participant 1, Makonde District, village health worker).*

Some of the rumours that circulated among people related COVID-19 vaccines to satanic origins. There were also reports that those who got vaccinated would change to monsters after 3 years. Thus, some people delayed vaccination since they wanted to observe the 3-year outcome for those who had taken the vaccine.

*“After some rumours of people changing into some monster after 3 years, people shunned vaccination” (FGD participant 6, Epiworth District, women leader)*.

In some places, people refused to be vaccinated based on the size of the needle as portrayed on the posters. They believed that the needle size was too large for human beings.

*“--- local people were not keen to be vaccinated based on the pictorial size of the injection on posters which people thought was meant for treating livestock” (FGD participant 9, Makonde District, village health worker).*

The phased approach implemented by the government in the COVID-19 vaccination programme raised fear and concern among the people, as they were not clear on the reasons behind the strategy. This was exacerbated when some government officials who had been among the first to be vaccinated died. Some individuals believed that this approach was being made because the vaccines were still under research.

*“Initially, when the COVID-19 vaccine was introduced, administration was done in phases starting with government officials followed by health workers, police and soldiers. This approach raised suspicion among the local people who viewed the COVID-19 vaccine as an experiment being imposed on the Zimbabwean population. This notion of the vaccine as being an experiment got supported in their thoughts as most government officials who were vaccinated first had an increase in death toll and this crippled people with fear” (FGD participant 2, Makonde District, village health worker).*

Changes made in the population age groups for vaccination also caused concern in some people. The youths thought that it was a drug to render them infertile.

*“The youths were not forthcoming as they said why initially we were not included among the vaccinated group. Why now, based on this, they viewed the vaccine as a drug to cause infertility and were so reluctant to accept it” (FGD respondent 3, Makonde District, village health worker)*.

Other people in the community thought that the position taken by the government to make vaccination optional in the first place when other countries were making it mandatory affected vaccine uptake. The change of policy from voluntary to mandatory vaccination, coupled with social media disinformation regarding the effects of the vaccine, led to the shunning of the COVID-19 vaccination programme.

*“-----the government at first did not take this practice seriously by making the vaccination optional. The time they started to enforce vaccination, this raised suspicion from the public coupled with social media misinformation, a lot of people declined vaccination giving so many reasons like it kills and so many unpleasant things. Those who had been vaccinated did not come for subsequent doses. ---” (FGD participant 1, Chiredzi District, traditional healer).*

## Discussion

The study has identified facilitators and barriers to vaccine uptake in Zimbabwe, thus providing contextual data about the specific needs and concerns of the Zimbabwean population regarding COVID-19 vaccines. It has provided future insights into areas that should be addressed in public health campaigns targeting vaccine uptake for emerging diseases. The issues raised in this study, although they may not be uniform in future epidemics, demonstrate a need for continued dialogue with the target population in the design and development of interventions for increased acceptability and success of interventions.

The study has shown that some individuals perceived the government-led vaccination programme to be the most preferred preventive measure for COVID-19, as they were hopeful that it would lead to herd immunity and the removal of non-pharmaceutical prevention measures. Confidence in government-led initiatives to curb COVID-19 in line with international guidelines has also been witnessed in Uganda [17]. Citizen trust in government increases adherence to public health interventions [18]. After having experienced more than 2 lockdowns and witnessed high case and death rates during the COVID-19 vaccination era, Zimbabwean citizens were now aware of the effects of the pandemic at the economic, social and family levels. Thus, preventive method that would allow them to go about their day-to-day business in coexistence with COVID-19 was now of priority. Studies elsewhere have also shown that people appreciate vaccines as a good public health measure for the prevention of the spread of COVID-19 [19, 20].

Similar to findings by Denford et al. [21], this study showed that some individuals did not believe that the COVID-19 vaccine was fully effective since they had encountered people who contracted the disease after being vaccinated. Some individuals were also not clear on why they should continue adhering to other COVID-19 preventive measures after vaccination, making them doubt the protective effect of the vaccine. In such circumstances, an explanation of the benefits of the vaccine should be coupled with an explanation on the population-level impact of the vaccine on COVID-19 transmission while ensuring that the message highlights both the benefits and limitations but is devoid of undermining public confidence in the vaccine.

The results showed that some Zimbabweans had witnessed COVID-19 vaccine side effects or were aware of them through interactions or media reports. They were also concerned about the accelerated development of the COVID-19 vaccines, perceiving that the production process lacked scientific rigour and long-term follow-up monitoring periods that would typically be employed in vaccine development. The continuously changing recommendations for vaccinations coupled with reports on social media regarding the discussions on the long-term effects of the vaccines among scientists and the social media reports affected citizens’ confidence in the vaccine and consequently compliance with vaccine uptake. It was the perception of participants that new information about the long-term safety of the COVID-19 vaccines may come to light at any point and preferred to wait until a certain time when the information was available before receiving the vaccine. Research studies conducted elsewhere during the COVID-19 pandemic have also documented supporting findings showing that vaccine hesitancy is the result of a lack of public confidence in the safety and efficacy of the vaccine and the risk and severity of COVID-19, among other factors [21–23]. However, because of mandatory vaccination policies, some people were vaccinated before they could observe the long-term effects of the vaccine and thus, may still be uneasy about what could happen to them. Recently, social media has been awash with some conditions that are perceived to be long-term effects of COVID-19. It is the responsibility of vaccine developers to monitor such situations and provide information that allays people’s fears regarding the unknown in the future. At country level, the government can make use of different communication channels such as media and trusted messengers to reach its people to dispel social media misinformation. Corroborating research in other settings [24–27], the participants in this study demonstrated that village health workers and community leaders are trusted messengers and that their participation in health programmes can result in increased uptake. It was revealed that some people started to accept COVID-19 vaccines after receiving health education from village health workers, health workers and motivation from community leaders.

The requirement for vaccination cards at ports of entry into the country and entry points into some premises, such as government entities, churches, or universities, had been enacted by the government to reduce transmission through immigration into other countries or emigration from other countries and locally in closed settings. However, this should be further explained so that population groups are provided with clarity of the policy to effect behaviour change.

Social media reported that the vaccine was a family planning method, resulting in infertility, continuous bleeding in women and reduced sexual activity in males. Such disinformation discouraged the reproductive age group from taking vaccines based on safety concerns. In such circumstances, even if individuals considered these side effects unlikely to occur, they would rather have COVID-19 than get vaccinated and risk suffering the presumed consequences. Findings elsewhere have shown that social media, conspiracy theories, and disinformation substantially increase hesitancy [28, 29]. Public health messaging should aim to address such issues to increase vaccine uptake. To ensure this and clear any misconceptions, there is a need for early community engagement [30]. As highlighted by Lucia et al. [31], there is a need for transparency and responses to concerns regarding the efficiency and safety of vaccine development. When the vaccination programme started in the country, the Ministry of Health and Child Care would monitor information from social media and counter it through public statements and press releases.

Participants cited lack of the vaccine at the health or vaccination centres as challenges affecting the accessibility of vaccines in their communities. When the vaccine were first introduced globally, it was estimated that it would take approximately seven months to produce enough vaccines to reach herd immunity by protecting 60–80% of the global population. The time indicated did not put into account other supply chain logistics such as transportation and administration procedures [32]. Thus, limited vaccine availability was a global barrier at the beginning of the COVID-19 vaccination programme. However, even when vaccine supply was increased, some populations could not access them due to lack of transport money or unavailability of transport for the health workers to bring the vaccines closer to the people. Such lack will usually affect the hard to reach areas and vulnerable groups including the elderly and poor who may not afford transport or have no transport means to the vaccination centres. Thus, even free pharmaceutical products need financial support for deployment within the reach of the affected populations [33, 34]. As recommended by Wouter et al. [33], in future pandemics, there is need to come up with early sustainable funding mechanisms for vaccines of emerging diseases to prevent distribution disparities of vaccines from global to community level.

The belief that the vaccine is a means to protect oneself from severe COVID-19 or death is consistent with previous research showing that the belief that health risk is likely or severe combined with the belief that one must use protection is known to be behaviour change motivators [35]. Since risk perception is dynamic, changing in response to disease situations and response to factors unrelated to the disease, it could have been more informative if a longitudinal assessment was made to interpret risk perception more accurately in the Zimbabwean setting.

Similar to findings in Bangladeshi [6], participants in this study showed that some individuals in Zimbabwe believed that mass vaccination would be the most effective approach to combating the COVID-19 pandemic; however, concerns regarding the side effects of the vaccine, vaccine effectiveness and safety delayed vaccine uptake by the population. Such concerns can lead to vaccine hesitancy. Additionally, delaying population-level protection is based on cultural and religious beliefs. To increase uptake in such populations, health promoters should aim to provide targeted messages to those population groups.

### Study limitations

The study was limited in that we collected data at a single point in time and thus could not observe the dynamism of facilitators and barriers to COVID-19 vaccine intake over time. While efforts were made to include a variety of social groups, the expressions documented here might not reflect the whole Zimbabwean population since the social groups were chosen from a few districts. Our study was also limited in that it did not include information on accessibility of health services and road networks in the study sites. Presentation of this information will have provided a nuanced understanding of the challenges and opportunities relating to healthcare access in our study sites. Another limitation of our study was that the study sites were chosen in districts with low coverage only, thus, we may have missed some of the facilitators to COVID-19 vaccination in Zimbabwe.

## Conclusion

The study has provided insights into social, behavioural and environmental factors leading to compliance with vaccine uptake in Zimbabwe. It showed that while in some instances, noncompliance is intentional, there are some nonintentional factors leading to poor compliance. It is thus important for health authorities to acknowledge and respond to these issues to improve vaccine uptake in an epidemic setting. Health authorities should also attach importance to improving risk communication and community engagement strategies to improve public trust in public health guidance.

## Data Availability

The audio recordings generated in this study are not available to the public for the sake of protecting participant confidentiality. Anonymized transcripts are available from the corresponding author upon reasonable request.

## Abbreviations

COVID–19: coronavirus disease 2019
DHIS2: District Health Information Software 2
FGD: Focus group discussion
KII: Key Informant Interview
